# Intelligence, Personality and Tolerance of Ambiguity

**DOI:** 10.3390/jintelligence11060102

**Published:** 2023-05-26

**Authors:** Stephen Cuppello, Luke Treglown, Adrian Furnham

**Affiliations:** 1 Department of Psychology, City University of London, London WCIE 7HX, UK; 2 Thomas International, Marlow SL7 1YG, UK; 3 Department of Leadership and Organizational Behaviour, Norwegian Business School (BI), Nydalveien, 0484 Oslo, Norway

**Keywords:** personality, intelligence, facets, compensation, investment

## Abstract

In this study, 3836 adults completed a personality test (the HPTI) and a multidimensional intelligence test (GIA). Two prominent theories that link personality traits to intelligence (compensation and investment) were tested. There were more sex differences in the personality traits than in the IQ scores. Correlational and regression analyses results provided little evidence for either theory but pointed to the role of tolerance of ambiguity as a consistently significant, positive correlate of IQ at both the facet and domain levels. The role of this neglected trait is discussed. Limitations of various aspects of this study and its implications are considered.

## 1. Introduction

There is longstanding interest in the relationship between personality and intelligence (Ackerman and Heggestad 1997; Bédard and Le Corff 2020; Eysenck 1998; Cattell 1971; Chamorro-Premuzic and Furnham 2004, 2006, 2008; DeYoung 2011, 2020; Kretzschmar et al. 2018; Major et al. 2014; Murray et al. 2014; Rammstedt et al. 2018; Reeve et al. 2006; Schermer et al. 2020; Zeidner and Matthews 2000; Ziegler et al. 2012). The relationship has been investigated in children and young adults (Johann and Karbach 2022) as well as in older adults (Gow et al. 2005). There is also an interesting and relevant literature linking intelligence with creativity (Corazza and Lubart 2021), dark-side traits (Lau et al. 2023), as well as examining how both personality and intelligence contribute to outcome measurements, such as work success (Macke et al. 2022).

Researchers have also developed theories regarding *why* certain traits (e.g., conscientiousness, openness) are related, albeit marginally, to intelligence (Chamorro-Premuzic and Furnham 2004; Rammstedt et al. 2018). The field has been reviewed (Anglim et al. 2022), and indeed there has been a special issue of this journal dedicated to the topic (Colom et al. 2019), as well as numerous other relevant papers (Demetriou et al. 2018; Willoughby et al. 2023).

There have been many differences in these studies with regard to the size and representativeness of the samples tested, as well as, perhaps more importantly, the nature of the tests used (Ackerman and Heggestad 1997; Furnham 2017a, 2017b; Furnham and Treglown 2018). It is not very easy to obtain a large, representative sample to test these hypotheses that can be traced over time, particularly using well known and robust intelligence tests, which often require up to an hour to complete (Beauducel et al. 2007; Gignac 2015; Johnson et al. 2008; Lohman and Lakin 2011). Further, as others have, Reeve and Blacksmith (2009) argued that an understanding of intelligence–personality associations requires the variance due to ‘g’ to be separated from the variance due to narrow cognitive abilities. Equally, it has been argued and demonstrated that it is important to examine the possibility of nonlinear relationships between the two variables.

### 1.1. Two Theories

There are two major theories in this area. First, *compensation theory* suggests that conscientiousness acts as a “coping/reimbursing strategy” for less intelligent, but ambitious and competitive, people in particular settings. Thus, *relatively* less intelligent individuals may become more methodical, organised, thorough, and persistent (i.e., conscientious) to compensate for their relative lack of intelligence in a highly competitive educational or work environment. That is, they can achieve as much as bright people by simply working harder and smarter. Alternatively, relatively more intelligent people may tend to succeed based on their cognitive efficiency, rather than strenuous effort or persistent effort and organisation.

There is some evidence for compensation theory. Moutafi et al. (2004) found conscientiousness to be more highly, but significantly negatively, correlated with fluid intelligence than crystallised intelligence, consistent with their theory. However, Wood and Englert (2009) found conscientious was negatively correlated with fluid *and* crystallised intelligence. Murray et al. (2014) argued and demonstrated that the true association between conscientiousness and IQ may be zero or positive at the population level but that the use of selected research samples has sometimes resulted in the appearance of a negative association. More recently, Harris-Watson et al. (2022) tested employee samples and found, in three of four samples, that the results supported a “nuanced compensatory mechanism”, showing that conscientiousness compensates for low to moderate GMA, but high conscientiousness may be detrimental to task performance in high-GMA individuals. Clearly, there remains much dispute over this theory.

von Stumm (2018) proposed an *investment theory* of adult intelligence, which posits that individual differences in knowledge attainment results from people’s differences in cognitive ability *and* their propensity to apply and invest that ability. These traits she referred to as *investment* personality traits. Thus, some traits, such as openness to experience, are related to IQ. von Stumm and Ackerman (2013) identified 34 trait constructs and corresponding scales that refer to intellectual investment, which were classified into different trait categories. These constructs included intellectual curiosity, abstract thinking, openness, absorption, ambiguity, novelty seeking, and social curiosity. In their meta-analysis of 112 studies with an overall sample of 60,097 participants, they found that investment traits were mostly positively associated with adult intellect markers ranging from 0 to .58, with an average estimate of .30. They concluded, however, that the strength of investment–intellect associations differs across trait scales and markers of intellect. In one study, Woods et al. (2019) found evidence for the theory using the California Personality Inventory (CPI).

Others have suggested that certain traits relate not so much to ability (IQ) but rather to the test-taking situation. For instance, it has been demonstrated that neuroticism is negatively correlated with both self-assessed and test-derived, IQ because test-anxiety influences performance (Furnham 2005). However, there have been a number of studies and theories to suggest that neuroticism is positively associated with academic success in highly selected groups (Austin et al. 2002; Leikas et al. 2009). However, this relationship is probably curvilinear since it is unlikely that high levels of anxiety could facilitate performance. Nevertheless, what is apparent is that the more “high stakes” that the test-taking situation is, the stronger that the relationship is between personality and intelligence. This point has been made by Major et al. (2014).

### 1.2. Other Traits

Although widely accepted, the Big Five do not encompass all personality traits that have been identified. Over the years, clinical, differential, and social psychologists have identified a large number of traits that could be logically and empirically related to intelligence. These traits include ones such as need for cognition and typical intellectual engagement (Furnham 2020). One recent study examined the relationship between strengths and fluid intelligence and found very little relationship except for the strength of “love of learning” (Kretzschmar et al. 2022).

This study explores two other traits that have been shown to relate to work performance.

The study used different, but validated, measures of both personality and intelligence. The High Potential Type Indicator (HPTI) was designed to assess personality in a work setting nearly 20 years ago. The idea was to assess those traits that had been demonstrated to be related to work performance. Initially, 12 traits were identified, but subsequent psychometric work identified six independent traits, which fulfilled the psychometric demands of a good test (Dissou 2003). A number of papers have used the HPTI (Furnham and Treglown 2018, 2021a, 2021b; Furnham and Impellizzeri 2021; Treglown and Furnham 2020, 2022). The psychometric properties of the measure have been reported (MacRae and Furnham 2020; Teodorescu et al. 2017). There is evidence of the construct, concurrent, and predictive validity of the measure, which has been used frequently in studies of businesspeople (Cuppello et al. 2023a, 2023b).

Four of the six HPTI scales are similar to components of the Big Five—conscientiousness, adjustment (low neuroticism), curiosity (openness), and competitiveness (low agreeableness). However, the HPTI does have two scales that measure concepts that have been part of the personality and individual difference literature for many years. Martinsen (2023) showed the concurrent validity of four dimensions when correlating HPTI and NEO-PI-R test scores in 1196 military people (correlation between adjustment and neuroticism were r = .62, that between curiosity and openness was r = .57, and that of both conscientiousness measures was r = .58.

The first, *ambiguity acceptance* (or tolerance of ambiguity; ToA) assesses how an individual or group processes and perceives unfamiliarity or incongruence. Those who are tolerant of ambiguity perform well in new or uncertain situations, adapt when duties or objectives are unclear, and are able to learn and function in unpredictable times or environments. The highly diverse literature has been reviewed by Furnham (2017a, 2017b) and Furnham and Marks (2013). Because studies have shown ToA to be correlated with openness (Caligiuri et al. 2000), as well as success in educational and work environments (Caulfield et al. 2014; Katsaros et al. 2014), we assumed that ToA would be positively correlated with intelligence.

The other variable was *approach to risk* or *courage,* which is the ability to combat or mitigate negative or threat-based emotions and broaden the potential range of responses. Unchecked fear restricts the potential range of responses and typically leads to behaviours such as avoidance or contrived ignorance, whereas courage is exhibited as the willingness to confront difficult situations and solve problems despite adversity. Courage is thought to be curvilinearly related to success in many work settings, with both high and low scorers being less successful. It is not clear whether courage would be related to intelligence.

We tested four hypotheses based on the previous literature: conscientiousness would be negatively correlated with overall intelligence (H1), curiosity (openness) would be positively correlated with overall intelligence (H2), adjustment (low neuroticism) would be positively correlated with intelligence (H3), and ToA would be positively correlated with overall intelligence (H4). We explored the relationships amongst the traits and the subscale scores on the IQ test but did not devise any specific hypotheses.

## 2. Method

### 2.1. Participants

There were 1380 women and 2456 men in our sample. Their average age was 38.57 years old (*SD* = 10.75). Nearly all were managers in various sectors, such as finance, technology, engineering, and human resources.

### 2.2. Measures

*The High Potential Traits Inventory* (HPTI) is a measure of normal, ‘bright’ personality traits, designed to ascertain how individuals think, prioritise, and act in the workplace (MacRae and Furnham 2020). The questionnaire comprises 78 items, with which participants decide the extent to which they agree on a 7-point Likert scale (1 = completely disagree to 7 = completely agree). Previous research has demonstrated that the HPTI assesses six dimensions of personality: conscientiousness, adjustment, curiosity, risk approach, ambiguity, and competitiveness.

*General Intelligence Assessment (GIA)*: The GIA was used to investigate gender differences in fluid intelligence. The GIA assesses individuals’ cognitive abilities by measuring their speed and accuracy across five domains relevant to work contexts—verbal reasoning, perceptual speed, number speed, word meaning, and spatial visualisation—as detailed in Table 1 (Dann 2015; Furnham and Treglown 2018). Its aim is to primarily measure mental speed of processing (i.e., fluid intelligence and procedural knowledge), rather than depth (i.e., crystallised intelligence and declarative knowledge). It reflects individuals’ ability to quickly process novel information and to learn, develop, and apply new skills (Dann 2015). The assessment consists of five tests (described in Table 1), which are developed in real time, via computer-based item generation. This method enables the automatic production of numerous different tests of equivalent form (Irvine et al. 1990). Each test measures a particular cognitive function and involves one type of task, and all questions within a test are of equal difficulty. The response format is multiple choice, and no time limit is imposed (Dann 2015).

### 2.3. Procedure

Participants were tested by a UK-based psychological consultancy. All data for this study were gathered from high-stakes testing for genuine occupational test use, including to inform recruitment processes, as part of employee development and the identification of high-potential employees within businesses. The participants took these and other tests as part of an assessment exercise run under strict guidelines, and the data were logged. Each participant was given personal detailed feedback about his or her score. The participants were nearly all employed as middle to senior managers in British companies. They agreed to their anonymised data being analysed and the results reported to further the understanding of assessment and selection. However, we had no more data than their sex, age, and occupational sector and when they were tested.

Structural equation modelling (SEM) was conducted in the Lavaan package of R (version 3.3.0). Since the data were not normally distributed, maximum likelihood with robust standard errors was used for parameter estimation. Based on Kline’s (2005) recommendations, the following fit indices were applied: RMSEA, standardised root mean residual (SRMR), comparative fit index (CFI), and the Tucker–Lewis index (TLI). An excellent fit is indicated when RMSEA < .05 (MacCallum et al. 1996), SRMR > .08 (Hu and Bentler 1998), CFI > .95 (Hooper et al. 2008), and TLI > .90 (Hu and Bentler 1998). Where chi-square statistics are reported, they represent the minimum function statistic corrected for non-normality, scaled by Satorra–Bentler factors.

## 3. Results

### Sex Differences

Because of the interest in sex differences, particularly in intelligence, a series of one-way ANOVAs were computed and are shown in Table 2. Considering results at the more conservative *p* < .001 level, there were four differences in personality with men scoring higher in conscientiousness, courage, ToA, and competitiveness. Similarly, half of the analyses for IQ showed a difference, with women scoring higher for reasoning but men scoring higher for numbers and spatial. However, the size of the differences was modest: e.g., risk approach (d = .36), competitiveness (d = .21), and numbers (d = .32).

## 4. Correlations

Table 3 shows the full correlation table, demonstrating that conscientiousness was only significantly correlated in one-sixth of the IQ scores and then negatively; curiosity was not significantly correlated with any of the IQ scores. Two of the correlations with neuroticism were significant, suggesting that those lower in adjustment performed better. However, all the correlations with ToA were significant, especially to the total score and words score (both *r* = .18).

## 5. Regressions

Next, a series of hierarchical regressions were performed with the total and subscale IQ scores being the criterion variables and demography and personality being the predictor variables. Age and sex were entered in the first step, followed by the six trait factors. The results are shown in Table 4.

In each regression, sex and age were significant and accounted for around 2% of the variance, with the exception of the number facet score, which accounted for 6% of the variance. Interestingly, the results were not fully consistent: in three analyses, men scored higher than women (total, numbers, spatial), but women performed better than men in two (reasoning, words). Similarly, younger people scored better than older people on all five analyses except for words.

There was a pattern also for the personality variables. For two, they were significant on all six analyses, showing that adjustment was negatively but ToA positively associated with the total IQ and subscores. Curiosity was a negative correlate in four and competitiveness in two, while courage was significant in one regressions and conscientiousness in none.

## 6. Structural Equation Model

SEM was used to explore the differentiating impact of tolerance to ambiguity on the relationship between personality and IQ. Three models were explored as a part of this analysis. The first was a holistic model in which IQ was entered as a latent variable, comprising five observed variables (i.e., the five subtests of the GIA: *Reasoning, Perceptual Speed, Number Speed, Word Meaning,* and *Spatial Visualisation*) and personality was entered as six observed variables (*conscientiousness, adjustment, curiosity, risk approach, ambiguity acceptance,* and *competitiveness*). In the second and third models, tolerance for ambiguity (*ambiguity acceptance*) was removed from the model and used to assess model fit for individuals with high (scores equal to or greater than the sample mean [49.64]) and low (scores less than the sample mean [49.64]).

**Figure 1:**
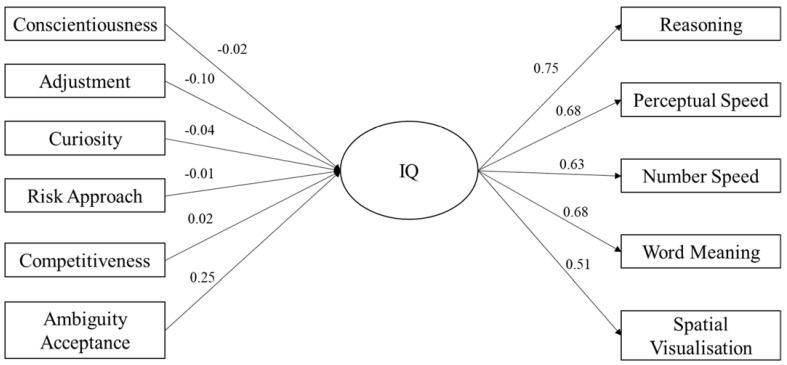
SEM showing relationship between personality and IQ.

Model 1 assessed the holistic impact of personality on IQ, the results of which can be seen in Figure 1. The model yielded a significant chi-square statistic (χ^2^(29) = 358.6, *p* < .001), indicating that the model deviates from the structure of the data. However, researchers have indicated that large sample sizes artificially inflate chi-square values, causing a rejection of the model. For this reason, other absolute fit indices were utilised, revealing an acceptable fit of the model: CFI = .94; TLI = .92; and RMSEA = .055 (90% CI upper limit = .060; 90% CI lower limit = .050). In model 1, ambiguity acceptance was the only significant, positive predictor (β = 0.25; *p* < .001), with adjustment (β = −.10; *p* < .001) and curiosity (β = −.04; *p* = .038) being significant, negative predictors of the latent IQ variable.

**Figure 2:**
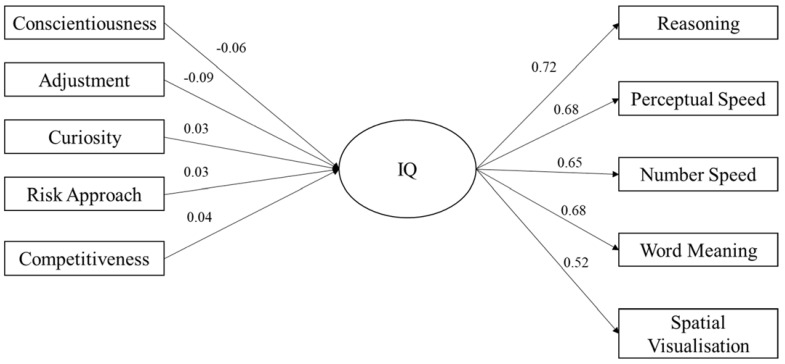
SEM showing relationship between personality and IQ for high tolerance of ambiguity.

Model 2 assessed the impact of personality on IQ for individuals with high tolerance for ambiguity, the results of which can be seen in Figure 2. The model yielded a significant chi-square statistic (χ^2^(25) = 194.7, *p* < .001). Other absolute fit indices revealed a good fit of the model: CFI = .93; TLI = .90; and RMSEA = .060 (90% CI upper limit = .072; 90% CI lower limit = .055), showing similar fit indices to those in model 1. In model 2, adjustment (β = −.09; *p* = .004) and conscientiousness (β = −.06; *p* = .047) were significant predictors of the latent IQ variable, differing slightly from those in model 1.

**Figure 3:**
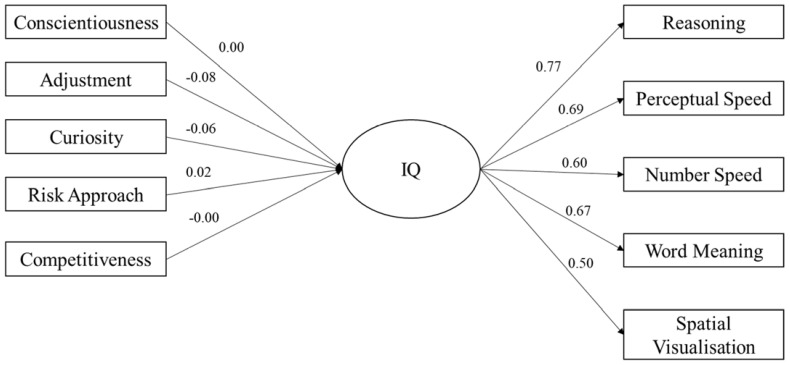
SEM showing relationship between personality and IQ for low tolerance of ambiguity.

Model 3 assessed the impact of personality on IQ for individuals with low tolerance for ambiguity, the results of which can be seen in Figure 3. The model yielded a significant chi-square statistic (χ^2^(35) = 146.2, *p* < .001). Other absolute fit indices revealed a good fit of the model: CFI = .95; TLI = .93; and RMSEA = .048 (90% CI upper limit = .056; 90% CI lower limit = .041), showing similar fit indices to those of models 1 and 2. In model 3, adjustment (β = −.08; *p* = .004) and curiosity (β = −.06; *p* = .003) were significant predictors of the latent IQ variable, showing similar predictors to those in model 1.

## 7. Discussion

The results of this study were particularly interesting for three reasons. First, there seemed to be no evidence for the *compensation* hypothesis, i.e., correlations between IQ and trait conscientiousness. Second, the results for the *investment* hypothesis went in the opposite direction: those who were high on curiosity/openness scored lower on IQ. Similarly, the two significant correlations with neuroticism went in the opposite direction, suggesting that those who were better adjusted performed worse overall on the IQ test. Third, the most clear, consistent, and significant correlate of the total IQ scale, as well as the subscales, was ToA.

The first issue consists of the nonsignificant or counterintuitive results. There may be a number of reasons for this outcome. The first is that neither the personality traits nor the intelligence test used here has been used to test this relationship. There is, however, evidence of concurrent validity with both tests (MacRae and Furnham 2020). It could be that the facets of conscientiousness, neuroticism, and openness, which are most closely related to intelligence, are not assessed by the HPTI, which does not have facet scores. 

However, what are perhaps more important is to understand are the participants and the testing situation. Nearly all the participants were middle-aged, mid-level managers being assessed for promotion or selection. Although all the scores were relatively normally distributed, they were skewed towards a more “positive profile”: higher scores on all six factors. Thus, this sample would be higher in both conscientiousness and openness, which could affect the results, bearing in mind that the relationship between personality and intelligence is always weak.

In all regressions, the adjustment score was a significant correlate, indicating that those who were better adjusted performed less well. The explanation for the finding might lie not so much in the tests as in the particular participants and the conditions under which the tests were taken. The participants were successful businesspeople with overall high adjustment scores. Furthermore, they took the test in a high-stakes situation, knowing that it had implications for selection and promotion. The data from students suggest that, amongst highly selected and more talented groups, neuroticism is positively, not negatively, related to academic success and IQ tests because anxiety results in greater effort.

However, by far, the most interesting finding was the association of ToA and IQ, indicating that the more ToA that a person was, the better that he or she performed on a test. Accepting ambiguity infers that there can consider multiple, even contradictory or combinatorial, solutions to problems. People with this mind-set could develop more sophisticated problem-solving techniques and interests, developing in turn higher fluid and crystallised intelligence. This relationship is related to second- or third-level intelligence test abilities being most closely linked to ToA. 

The results are seen more clearly in the SEM. Ambiguity acceptance was the only significant, positive predictor of the latent IQ variable, with adjustment and curiosity being significant, negative predictors.

In a recent comprehensive review of ToA, de Vries (2021) noted the many related concepts of ToA, which include “epistemic curiosity”, “curiosity and exploration inventory”, “need for cognition”, “openness for ideas”, “typical intellectual engagement”, “need for structure”, “need for precision”, and “need for evaluation”, as well as “dialectical” and “paradoxical” thinking. The idea is that the trait of ToA leads to behaviours such as the need to know more and to understand the environment, which logically seem related to intelligence. Many of these concepts have measures that have been shown to be related to ToA (Furnham 2020).

Certainly, this study has emphasized the role of a new trait as it relates to intelligence. Indeed, this emphasis might complement compensation and investment theory and be considered something akin to clarification theory: the idea that ToA encourages the act of trying to understand complexity, which relates to learning.

The results from the regression on total IQ in Table 3 confirm much of what we know about IQ. Thus, comparatively older people performed less well on most tests, particularly reasoning, but better on words/vocabulary, reflecting the well-established difference between fluid and crystallised intelligence. Similarly, the gender differences showed that men scored higher on spatial and number scales, but women scored better on reasoning tasks.

All researchers who have investigated the association between personality and intelligence have accepted that, whilst there are theoretical reasons to suppose relationships, they are not strong (i.e., have small effect sizes) and can be influenced by the particular tests used, as well as the testing situation (DeYoung 2011, 2020). In this study, we had a select group (mainly middle-aged businesspeople) completing less well-known tests in a selection environment. The results most of all have highlighted a variable that has been less explored in the personality-intelligence literature and that deserves greater attention.

## Figures and Tables

**Table 1:** GIA Tests’ Descriptions.

Test	Description	Format	Length (Minutes)	Abilities Tested
VR	Evaluates problem-solving abilities (i.e., capacity to reason, make inferences, draw conclusions) by testing simple deductive verbal reasoning skills	Problem-solving task: After reading a statement (e.g., Jack is taller than Jill), participants need to answer a related question (e.g., Who is shorter? Jack or Jill?).	5	Fluid and crystallised intelligence
PS	Measures visual checking skills (i.e., ability to identify and report on similarities/differences, details, and errors) by testing semantic perception and encoding	Letter-matching task: Participants need to identify matching letters between rows of capital and lower case letters (e.g., ADGK/afgm).	4.5	Broad cognitive speed
NS	Assesses overall numeracy (i.e., capacity to process numerical information, perform mental calculations, and reason with quantitative concepts)	Number task: Out of three numbers, participants need to identify which number is numerically farther from the others (e.g., 2, 9, 5).	2	Fluid intelligence and memory
WM	Evaluates vocabulary and word-related knowledge (i.e., ability to comprehend large numbers of words and identify words with similar or opposite meanings)	Semantic word task: Participants are shown three words (e.g., up, down, street) and need to specify which word is not related to the others (e.g., street).	2.5	Mainly crystallised intelligence
SV	Tests mental visualisation skills (i.e., ability to visualise concepts and objects and mentally rotate and manipulate shapes and symbols)	Symbol task: Participants need to identify pairs of identical symbols (when symbols have been rotated and/or presented as mirror images of each other).	2	Fluid intelligence and visual perception

*Note.* VR = verbal reasoning; PS = perceptual speed; NS = number speed; WM = word meaning; SV = spatial visualisation.

**Table 2:** Sex Differences in Personality and IQ Factors.

		M	SD	F	Cohen’s d
Conscientiousness	Female	65.46	15.69	14.11	−.13
	Male	67.41	15.29		
Adjustment	Female	64.89	16.28	5.50	−.08
	Male	66.15	15.71		
Curiosity	Female	60.84	13.16	1.12	.04
	Male	60.39	12.43		
Risk Approach	Female	58.15	13.35	108.44	−.35
	Male	62.68	12.68		
Ambiguity Acceptance	Female	48.49	12.10	20.20	−.15
	Male	50.29	11.85		
Competitiveness	Female	46.96	13.80	67.84	−.28
	Male	50.68	13.18		
Overall IQ	Female	51.05	11.02	5.06	−.08
	Male	51.92	11.86		
Reasoning	Female	40.77	8.77	22.02	.16
	Male	39.36	9.08		
Perceptual	Female	43.02	6.55	2.20	.05
	Male	42.69	6.59		
Numbers	Female	13.36	5.53	85.75	−.32
	Male	15.26	6.42		
Words	Female	30.31	5.61	1.65	.04
	Male	30.07	5.68		
Spatial	Female	9.57	5.10	65.26	−.27
	Male	10.98	5.22		

**Table 3:** Descriptive Statistics and Pearson’s Correlations between Demographic, Personality, and IQ.

	M	SD	1	2	3	4	5	6	7	8	9	10	11	12	13	14
1. Gender	1.64	.48														
2. Age	38.57	10.75	.19 ***													
3. Conscient.	66.71	15.46	.06 ***	.05 **												
4. Adjust.	65.70	15.93	.04 *	.07 ***	.41 ***											
5. Curiosity	60.55	12.70	−.02	−.05 **	.30 ***	.21 ***										
6. Risk Appro.	61.05	13.11	.17 ***	.12 ***	.54 ***	.45 ***	.40 ***									
7. Amb. Accept.	49.64	11.97	.07 ***	.15 ***	.15 ***	.33 ***	.31 ***	.37 ***								
8. Competitive.	49.34	13.52	.13 ***	−.21 ***	.31 ***	−.03 *	.11 ***	.27 ***	.00							
9. Overall IQ	51.61	11.58	.04 *	−.12 ***	**−.03**	−.04 *	**.01**	**.02**	.18 ***	**.02**						
10. Reason	39.87	9.00	−.08 ***	−.16 ***	**−.03**	**−.02**	**.00**	**−.01**	.14 ***	**.00**	.79 ***					
11. Percept	42.81	6.58	−.02	−.15 ***	**−.00**	**.00**	**.01**	.03 *	.09 ***	**.03**	.77 ***	.52 ***				
12. Number	14.58	6.18	.15 ***	−.16 ***	**−.01**	**−.03**	**−.02**	**.02**	.14 ***	.10 ***	.73 ***	.45 ***	.42 ***			
13. Words	30.16	5.66	−.02	.09 ***	−.05 **	−.07 ***	**.02**	**−.01**	.18 ***	−.08 ***	.74 ***	.55 ***	.46 ***	.39 ***		
14. Spatial	10.47	5.22	.13 ***	−.03	**−.00**	**−.02**	**.02**	.04 **	.11 ***	**.01**	.63 ***	.34 ***	.37 ***	.41 ***	.32 ***	

*** *p* < .001, ** *p* < .01, * *p* < .05.

**Table 4:** Results for Regression of IQ Scores on Demographics and Traits.

	Overall IQ					Reasoning						Numbers				
	*B*	*SE*		*Beta*	*t*	*B*		*SE*	*Beta*	*t*		*B*	*SE*	*Beta*		*t*
Gender	1.27	.39		.05	3.23 **	−.99		.31	−.05	−3.23 **		2.19	.21	.17		10.60 ***
Age	−.18	.02		−.17	−9.92 ***	−.16		.01	−.19	−11.02 ***		−.12	.01	−.21		−12.88 ***
Conscient.	.00	.02		.00	0.04	−.00		.01	−.00	−0.10		.00	.01	.01		0.33
Adjust.	−.08	.01		−.11	−5.60 ***	−.04		.01	−.06	−3.38 ***		−.03	.01	−.07		−3.84 ***
Curiosity	−.05	.02		−.06	−3.30 ***	−.04		.01	−.06	−3.40 ***		−.04	.01	−.08		−4.35 ***
Risk Appro.	.02	.02		.02	0.99	.01		.02	.01	0.49		−.00	.01	−.01		−0.45
Amb. Accept.	.23	.02		.24	13.67 ***	.15		.01	.20	11.48 ***		.11	.01	.21		11.87 ***
Competitive.	−.02	.02		−.03	−1.55	−.02		.01	−.03	−1.51		.02	.01	.04		2.01
Adjusted *R*^2^	.066					.061						.093				
*F*	34.815					32.058						50.131				
*p*	.000					.000						.000				
	Words								Spatial							
	*B*		*SE*		*Beta*		*t*		*B*		*SE*		*Beta*		*t*	
Gender	−.40		.19		−.03		−2.08 *		1.49		.18		.14		8.26 ***	
Age	.03		.01		.06		3.59 ***		−.04		.01		−.08		−4.70 ***	
Conscient.	−.00		.01		−.00		−0.11		.00		.01		.01		0.41	
Adjust.	−.05		.01		−.14		−7.54 ***		−.03		.01		−.08		−4.18 ***	
Curiosity	−.01		.01		−.01		−0.59		−.01		.01		−.02		−0.97	
Risk Appro.	−.00		.01		−.01		−0.34		.01		.01		.03		1.44	
Amb. Accept.	.11		.01		.23		12.90 ***		.06		.01		.13		7.33 ***	
Competitive.	−.03		.01		−.06		−3.37 ***		−.02		.01		−.04		−2.12 *	
Adjusted *R*^2^	.061								.035							
*F*	31.911								18.446							
*p*	.000								.000							

*** *p* < .001, ** *p* < .01, * *p* < .05.

## Data Availability

Data available from the first author.
